# Supplementary material for: Nonreciprocal charge transport up to room temperature in bulk Rashba semiconductor α-GeTe
Source: Nat Commun. 2021 Jan 22;12:540. doi: 10.1038/s41467-020-20840-7 (PMC7822853; doi:10.1038/s41467-020-20840-7)
Supplement: Supplementary file 1 — Supplementary Information [file 41467_2020_20840_MOESM1_ESM.pdf]

SUPPLEMENTARY INFORMATION

Nonreciprocal charge transport up to room temperature in bulk Rashba semiconductor α-GeTe

Yan Li[1], Yang Li[2,3], Peng Li[1], Bin Fang[1], Xu Yang[2,3], Yan Wen[1], Dong-xing Zheng[1], Chen-hui Zhang[1], Xin He[1], Aurélien Manchon[1,4], Zhao-Hua Cheng[2,3,5*], and Xi-xiang Zhang[1*]

[1]Physical Science and Engineering Division, King Abdullah University of Science and Technology (KAUST), Thuwal 23955–6900, Saudi Arabia

[2]State Key Laboratory of Magnetism and Beijing National Laboratory for Condensed Matter Physics, Institute of Physics, Chinese Academy of Sciences, Beijing 100190, China

[3]School of Physical Sciences, University of Chinese Academy of Sciences, Beijing 100049, China

[4]Aix-Marseille Univ, CNRS, CINaM, Marseille, France

[5]Songshan Lake Materials Laboratory, Dongguan, Guangdong 523808, China

These authors contributed equally: Yan Li, Yang Li.

[*]Corresponding authors:

Xi-xiang Zhang (email: xixiang.zhang@kaust.edu.sa)

Zhao-Hua Cheng (email: zhcheng@iphy.ac.cn)

Supplementary Note 1: Method details of ac harmonic measurements

When applying an electric field E_x, the charge current density can be expanded as

$$J_x = J_x^1 + J_x^2 = \sigma_1 E_x + \sigma_2 E_x^2 \quad \text{(S1)}$$

Here, σ_1 and σ_2 are first- and second-order conductance, respectively.

In the case of $\sigma_2 E_x^2 \ll \sigma_1 E_x$, the longitudinal resistivity writes as[1],

$$\rho_{xx} = \frac{E_x}{J_x} = \frac{1}{\sigma_1 + \sigma_2 E_x} \approx \frac{1}{\sigma_1} - \frac{\sigma_2 E_x}{\sigma_1^2} \quad \text{(S2)}$$

Considering the expression of the nonreciprocal charge transport $R(\boldsymbol{B}, \boldsymbol{I}) = R_0(1 + \gamma \boldsymbol{B} \cdot \boldsymbol{I})$, Eq. S2 can give out,

$$\gamma = -\frac{\sigma_2}{wdB_y\sigma_1^2} \quad \text{(S3)}$$

$$R_0 = \frac{l}{wd\sigma_1} \quad \text{(S4)}$$

Here, *w*, *l* and *d* are the length, width and thickness of the Hall bar, respectively.

AC harmonic measurement technique is employed to detect the nonreciprocal charge transport in α-GeTe. Injecting a sinusoidal ac current $I_\omega = I_0 \sin\omega t$ into the device along x axis, the longitudinal voltage writes as[2],

$$V(t) = I_\omega R = R_0 I_0 \sin\omega t + \gamma R_0 B I_0^2 \cos\varphi \sin^2\omega t$$

$$= R_0 I_0 \sin\omega t + \frac{1}{2}\gamma R_0 B I_0^2 \cos\varphi [1 + \sin\left(2\omega t - \frac{\pi}{2}\right)] \quad \text{(S5)}$$

It is seen from Eq. S5 that the first and second harmonic signals are given by

$$V_\omega(t) = R_0 I_0 \sin\omega t \quad \text{(S6)}$$

$$V_{2\omega}(t) = \frac{1}{2}\gamma R_0 B I_0^2 \cos\varphi \sin\left(2\omega t - \frac{\pi}{2}\right) \quad \text{(S7)}$$

Therefore, the second-order harmonic resistance $R_{2\omega}$ is expressed as,

$$R_{2\omega} = \frac{V_{2\omega}}{I_0} = \frac{1}{2}\gamma R_0 B I_0 \cos\varphi \quad \text{(S8)}$$

The amplitude of $R_{2\omega}$ is extracted as,

$$\Delta R_{2\omega} = \frac{1}{2}\gamma R_0 B_y I_0 \tag{S9}$$

The combination of Eq. S3 and Eq. S9 give $\Delta R_{2\omega} \propto \sigma_2$ or $J_x^2 \propto \Delta R_{2\omega}$ at a fixed E_x.

Supplementary Note 2: Calculation of the nonreciprocal charge transport

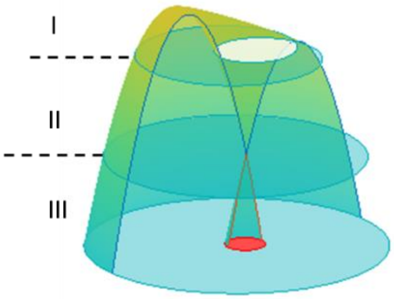

Supplementary Fig. 1 Illustrations of the Rashba-type band structure. Rashba-type band structure with in-plane magnetic field and shape of the Fermi surface at varied μ.

In 2D momentum space (k_x, k_y), the corresponding Hamiltonian with in-plane magnetic field B_y along y-axis can be expressed as[2]

$$H = \frac{\hbar(k_x^2+k_y^2)}{2m^*} + \alpha(k_x\sigma_y - k_y\sigma_x) - \frac{1}{2}g\mu_B B_y\sigma_y \tag{S10}$$

Here, $\hbar$, α , m^*, g =57 (assuming the same value as SnTe[3]) and μ_B are the Planck's constant, Rashba parameter, effective mass of electrons, Landé g-factor and Bohr magneton, respectively. σ_x and σ_y are the Pauli matrices. Eigenvalues of the Hamiltonian correspond to the two spectral branches,

$$\varepsilon(\mathbf{k}) = \frac{\hbar^2(k_x^2+k_y^2)}{2m^*} \pm \alpha\sqrt{(k_x - \frac{g\mu_B B_y}{2\alpha})^2 + k_y^2} \tag{S11}$$

The total charge current density is given by the following integral,

$$J_x = J_x^1 + J_x^2 = e\int \frac{d^2\mathbf{k}}{(2\pi)^2} v(\mathbf{k})[f_1(\mathbf{k}) + f_2(\mathbf{k})] \tag{S12}$$

Here, $v(\mathbf{k}) = \partial\varepsilon(\mathbf{k})/\hbar\,\partial\mathbf{k}$, f_1 and f_2 are the carrier group velocity, first and second order occupation functions, respectively. According to Boltzmann equation, f_1 and f_2 can be described using the Fermi-Dirac distribution f_0 as

$$f_1 = \frac{e\tau E_x}{\hbar}\frac{\partial f_0}{\partial \mathbf{k}} \tag{S13}$$

$$f_2 = \frac{e^2\tau^2 E_x^2}{\hbar^2}\frac{\partial^2 f_0}{\partial \mathbf{k}^2} \tag{S14}$$

Here, τ and E are the scattering time and electric field. As seen above, the first and second charge currents depend on the terms weighed by f_1 and f_2. Supplementary Fig. 2 shows the magnetic field dependence of the second-order charge current J_x^2. It is found that the second order current is odd function about the applied magnetic field, i.e., $J_x^2(-B_y) = -J_x^2(B_y)$. Moreover, it is noteworthy that the characteristics of the nonreciprocal charge transport with temperature and Fermi level position are also directly influenced by the types of carriers considering that the parabolic curves of the bulk Rashba-type bands point downwards or upwards. The nonreciprocal response up to room temperature strongly benefits from its giant bulk Rashba spin splitting energy and stable noncentrosymmetric structure.

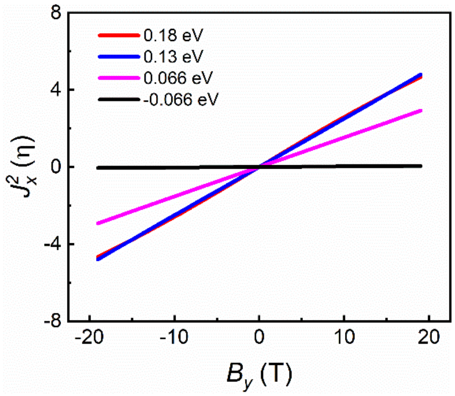

Supplementary Fig. 2 The calculated second-order charge current J_x^2. J_x^2 (in unit of $\eta = \frac{e^3\tau^2}{2m^*\alpha\hbar^2 E_x^2}$) as functions of the applied magnetic field B_y at different Fermi level position at 200 K.

In the text, we discuss a 2D Rashba to state the negligible contribution of the surface Rashba states to the nonreciprocal charge transport in α-GeTe. Here, the 2D model is extended to the bulk Rashba systems. The Hamiltonian with B_y along y-axis in 3D Rashba system reads as[2],

$$H = \frac{\hbar(k_x^2+k_y^2)}{2m_\perp} + \frac{\hbar k_z^2}{2m_\parallel} + \alpha(k_x\sigma_y - k_y\sigma_x) - \frac{1}{2}g\mu_B B_y\sigma_y \tag{S15}$$

Eigenvalues of the corresponding Hamiltonian writes as,

$$\varepsilon(\mathbf{k}) = \frac{\hbar^2(k_x^2+k_y^2)}{2m_\perp} + \frac{\hbar^2 k_z^2}{2m_\parallel} \pm \alpha\sqrt{(k_x - \frac{g\mu_B B_y}{2\alpha})^2 + k_y^2} \tag{S16}$$

We can follow the calculation in 2D model via setting

$$\mu \to \mu - \frac{\hbar^2 k_z^2}{2m_\parallel} \tag{S17}$$

The corresponding first- $J^1_{x,3D}$ and second-order charge current $J^2_{x,3D}$ is given by the following integral[2],

$$J^1_{x,3D} = \int J^1_x(k_z)\frac{dk_z}{2\pi} \tag{S18}$$

$$J^2_{x,3D} = \int J^2_x(k_z)\frac{dk_z}{2\pi} \tag{S19}$$

Even though $J^2_{x,3D}$ is nonzero below DCP considering the integral in Eq. S(19), the nonreciprocal coefficient γ ($\propto \frac{J^2_{x,3D}}{(J^1_{x,3D})^2}$) below DCP is relatively small, as shown in Supplementary Fig. 3. These suggest the validity of the nonreciprocal charge transport model in 3D Rashba systems. For the sake of simplicity, we only discuss a 2D Rashba model in the text.

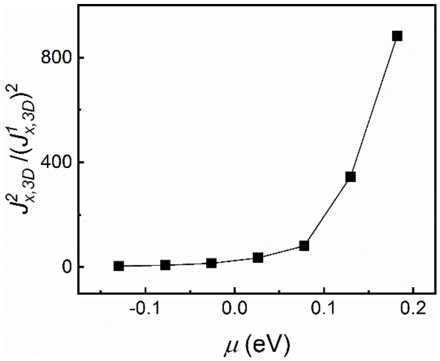

Supplementary Fig. 3 The calculated nonreciprocal coefficient γ. γ versus the Fermi level position μ in 3D Rashba systems.

Supplementary Note 3: Sample characterizations

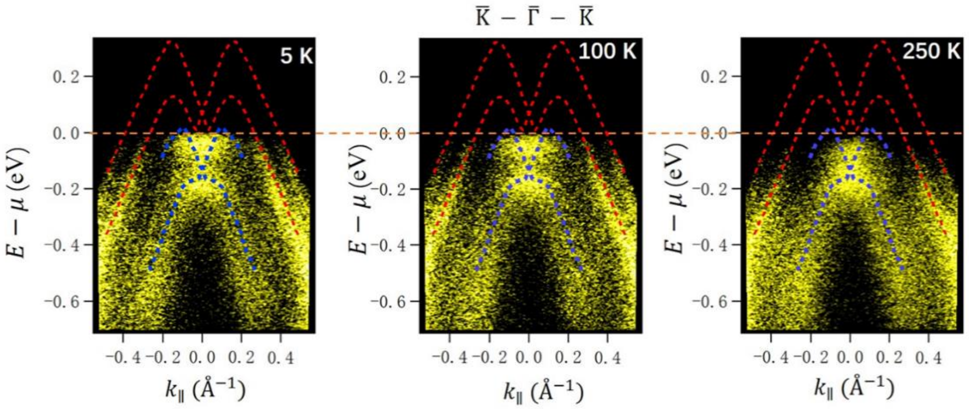

Supplementary Fig. 4 ARPES measurements at various temperatures. Temperature evolution of the band structures along $\bar{K} - \bar{\Gamma} - \bar{K}$ direction in α-GeTe/Al_2O_3(0001).

In order to further confirm the evolution of the Fermi level and the Rashba constant with temperature, we carried out the ARPES measurements on α-GeTe at varied temperatures. It is seen that the Fermi level position and the Rashba constant in α-GeTe keep almost unchanged with varying temperature, as shown in Supplementary Fig. 4, which is consistent with the recent report[4]. This is also in consistent with the fact that the absolute change in the temperature dependent carrier concentration remains weak in the magnitude, as shown in Fig. 1(d).

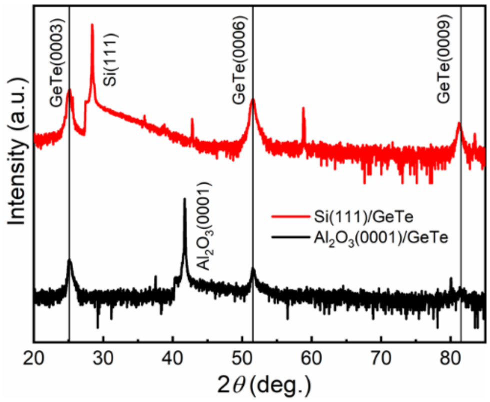

Supplementary Fig. 5 XRD measurements. XRD patterns of α-GeTe films on substrates Al_2O_3(0001) and Si(111).

Supplementary Fig. 5 shows X-ray diffraction (XRD) patterns of α-GeTe films deposited on Al_2O_3(0001) substrates in comparison with those grown on Si(111) substrates. As only three (000l) diffraction peaks of the trigonal α-GeTe structureas are present, indicating that the GeTe grew exclusively with well-ordered out-of-plane orientation.

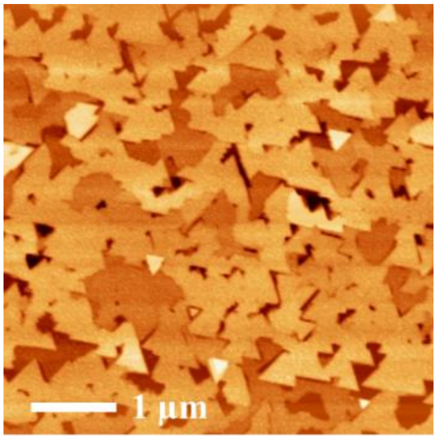

Supplementary Fig. 6 AFM measurement. AFM image of α-GeTe.

Supplementary Fig. 6 show atomic force microscope (AFM) image of α-GeTe with classic triangles features.

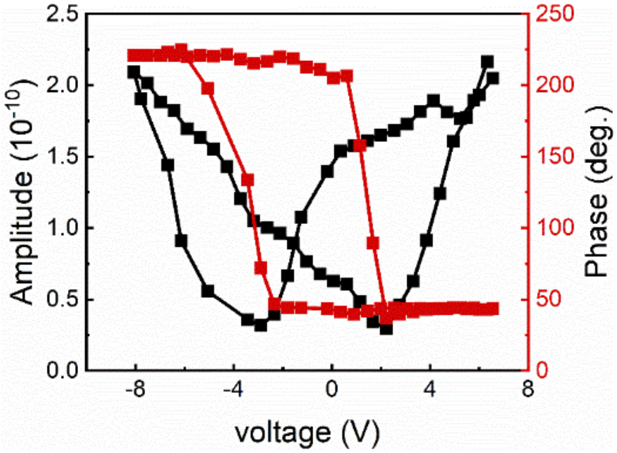

Supplementary Fig. 7 PFM measurement. PFM amplitude hysteresis loops and phase as a function of dc tip voltage.

The domain structure was detected by a piezoelectric force microscope (PFM). Supplementary Fig. 7 shows the PFM phase hysteresis loop, indicating the reversal of the polarization direction in α-GeTe films induced by the electric field. The characteristic “butterfly” shape of the PFM amplitude as a function of dc tip voltage is also observed, thus corresponding exactly to the ferroelectric domain switching behavior of the GeTe films.

Supplementary Note 4: Measurements of charge transport

As shown in Supplementary Fig. 8, angular dependent first- (R_{ω}) and second-order harmonic resistances ($R_{2\omega}$) in α-GeTe hold the period of 180° and 360°, respectively.

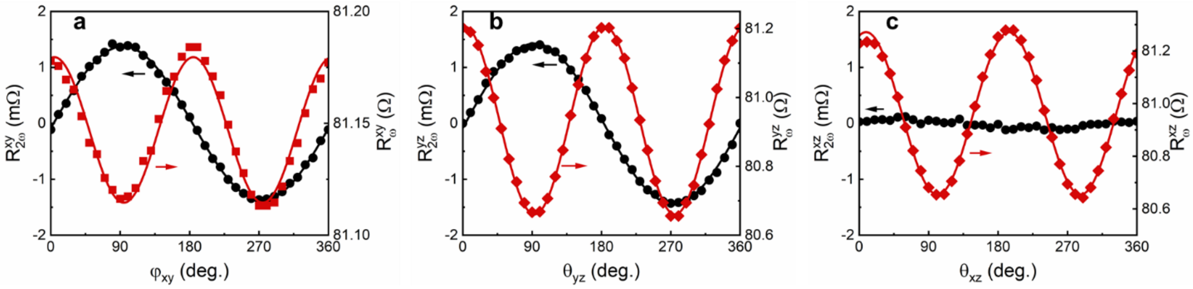

Supplementary Fig. 8 First- and second-order harmonic longitudinal resistances in three different geometries. Angular dependent first- (R_{ω}) and second-order ($R_{2\omega}$) harmonic longitudinal resistances with the current density $j = 7.5 \times 10^{5}\ Acm^{-2}$ and magnetic field B = 9 T

at T = 10 K in the α-GeTe with a thickness of 64 nm (sample 1) during rotating the magnetic field in the xy (**a**), yz (**b**), and xz (**c**) planes.

To identify the contribution of the thermoelectric effect (Nernst effect) to the second-order harmonic resistance, the angular dependent second-order harmonic transverse (R_{xy}) resistance is also measured, as shown in Supplementary Fig. 9. It is seen that $R_{2\omega}^{xy}$ doesn't nearly manifest cosφ or sinφ behaviors with $R_{2\omega}^{xy}/R_{2\omega}^{xx} \ll w/l$. Therefore, we can neglect the contribution of the thermoelectric effect to the measured second-order harmonic longitudinal resistance.

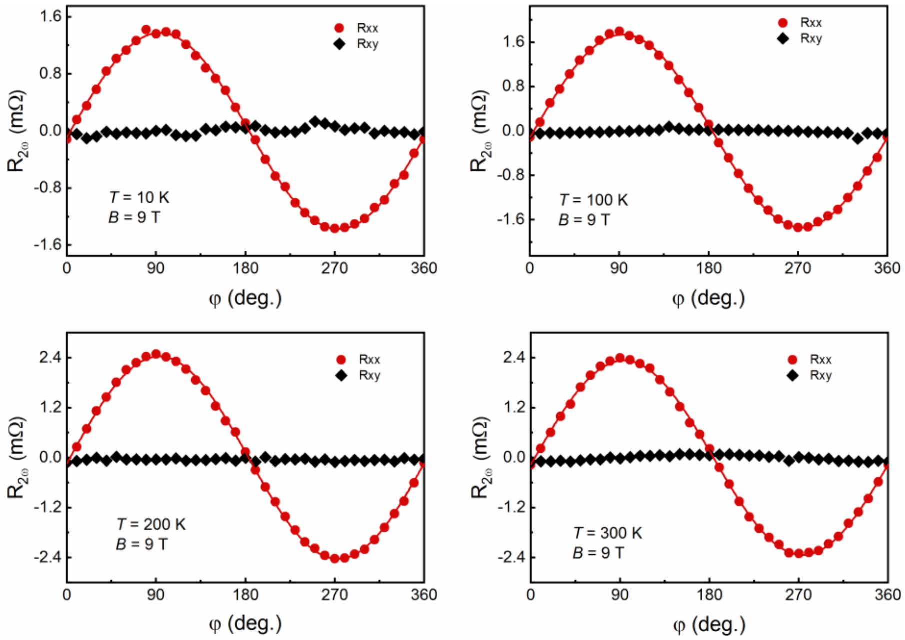

Supplementary Fig. 9 Thermoelectric effect. Angular dependent second-order harmonic longitudinal (R_{xx}) and transverse (R_{xy}) resistances with the current density $j = 7.5 \times 10^5\ Acm^{-2}$ and magnetic field B = 9 T at different temperatures in the α-GeTe with a thickness of 64 nm (sample 1) during rotating the magnetic field in the xy plane.

For convenience, we refer to the α-GeTe film with a thickness of 64 nm in main text. In reality, similar experimental results can be also observed in other α-GeTe films with different thickness. In the following, we show our observations in the α-GeTe with a thickness of 40 nm (sample 2) as an example, as shown in Supplementary Fig. 10-13.

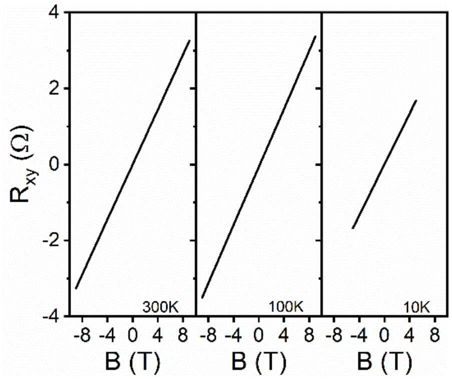

Supplementary Fig. 10 Measurements of carrier concentration. Magnetic field dependence of the Hall resistance R_{xy} at 300 K, 100 K and 10 K in the α-GeTe with a thickness of 40 nm (sample 2).

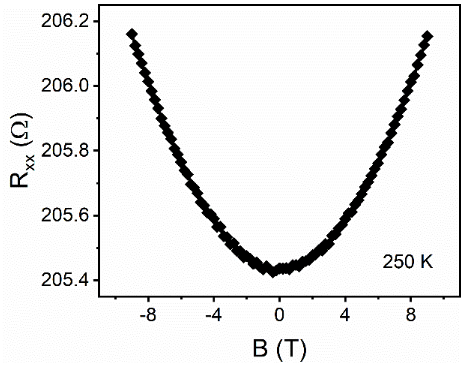

Supplementary Fig. 11 Magnetoresistance at different magnetic fields. Magnetic field dependence of the longitudinal resistance R_{xx} at 250 K in the α-GeTe with a thickness of 40 nm (sample 2).

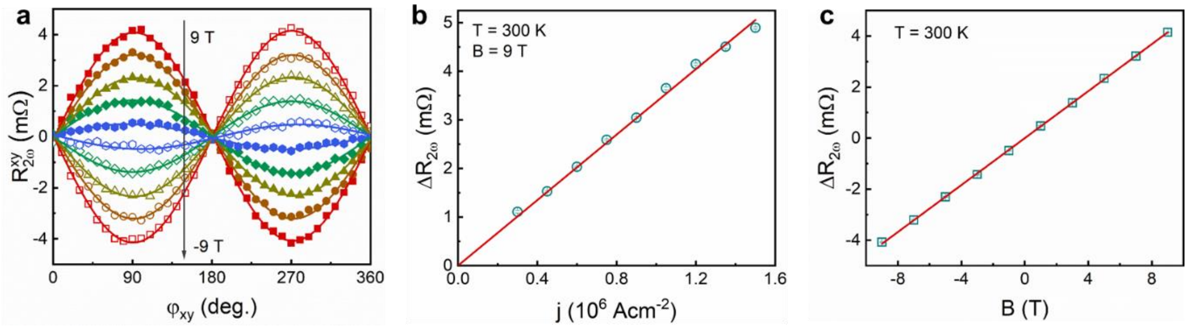

Supplementary Fig. 12 Nonreciprocal charge transport in α-GeTe (sample 2). a Angle-dependent second-order harmonic longitudinal resistance ($R_{2\omega}$) at varied magnetic fields at 300 K in the α-GeTe with a thickness of 40 nm (sample 2). **b** $\Delta R_{2\omega}$ as a function of the applied ac current

amplitude at 300 K and 9 T. **c** The magnetic field dependent $\Delta R_{2\omega}$ extracted from **a** with $j = 1.2 \times 10^{6}\ Acm^{-2}$ at T= 300 K.

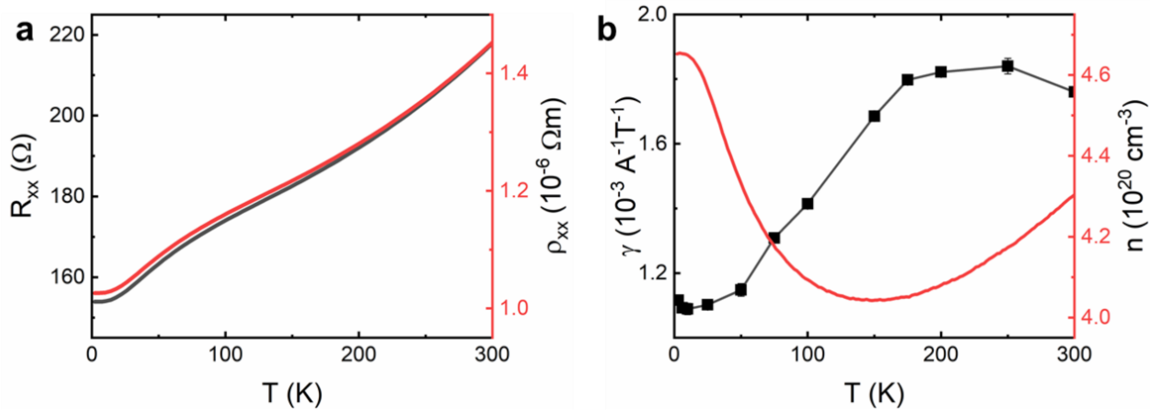

Supplementary Fig. 13 Electric transport in α-GeTe (sample 2). a Temperature dependences of the longitudinal resistance R_{xx} and resistivity ρ_{xx} in the α-GeTe with a thickness of 40 nm (sample 2). **b** Evolution of the nonreciprocal coefficient γ and the carrier concentration n with temperature.

We further performed the measurements of the nonreciprocal charge transport above 300 K using the PPMS system. As shown in Supplementary Fig. 14a, the nonreciprocal transport behavior in α-GeTe is still evident at 375 K. In addition, the γ vs. T curve still follows the same trend at a temperature much high than room temperature, as shown in Supplementary Fig. 14b.

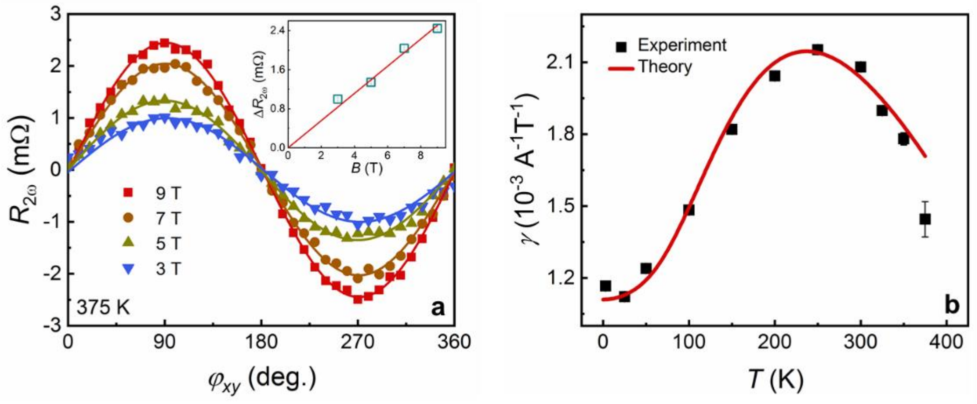

Supplementary Fig. 14 Nonreciprocal charge transport in α-GeTe (sample 3). a In-plane angle-dependent second-order harmonic longitudinal resistance ($R_{2\omega}$) at various magnetic fields in α-GeTe with a thickness of 40 nm (sample 3). The inset is the extracted magnetic field dependent

$\Delta R_{2\omega}$. **b** The experimental data of temperature dependent nonreciprocal coefficient γ are plotted with the theoretical prediction.

We further carried out the harmonic measurements in Hall bar devices covered with different layers [bare, 2 nm TiO_2 (sample 4) and 3 nm SiO_2 (sample 3)]. It is clearly seen in Supplementary Fig. 15 that up to the room temperature, the nonreciprocal charge transport characteristic (the non-monotonical γ vs. T curves) in α-GeTe is independent of the properties of the surface, such as, covered by air, TiO_2 and SiO_2. The results further support our conclusion that the nonreciprocal transport arises from bulk Rashba spin-orbit coupling rather than the surface Rashba states.

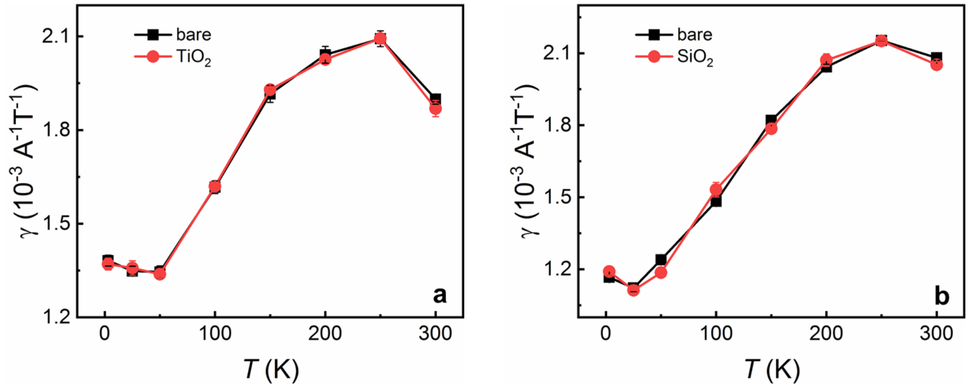

Supplementary Fig. 15 Nonreciprocal charge transport in α-GeTe with different surface cappings. Evolution of the nonreciprocal coefficient γ with temperature in α-GeTe with **a** TiO_2 (sample 4) and **b** SiO_2 (sample 3) surfaces.

For the epitaxial α-GeTe(0001) film [*termed as α-GeTe(111) in Rhombohedral cell choice, α-GeTe(0001) in Hexagonal cell choice*[5]], there is a preferential ferroelectric order with the distortion axis along *c*-axis. It is reported that the epitaxial α-GeTe film grown via MBE technique is single domain over several hundred μm^2, and the polarity can be sustained over the thickness of several hundred nanometers[6-8]. This stable domain is present everywhere in the films and can only be interrupted by stripe like domains with oblique distortion axis, which is confirmed by the fact that this structure does not randomize the ferroelectric order[8]. Meanwhile, the self-screening process of free carriers provided by intrinsic Ge vacancies in GeTe films cancels the depolarization field according to first principles calculations and results in a pronounced stability of ferroelectricity in thin films α-GeTe[9,10]. Therefore, the used Hall bar devices with 5 μm × 30 μm × 64 nm in the main text can be regarded as single domain. To further verify our claim, we have also measured

the nonreciprocal charge transport in the devices fabricated using the same film but with a much smaller size of 0.5 μm × 1.5 μm × 64 nm via the e-beam lithography, as shown in Supplementary Fig. 16. The nonreciprocal charge transport has been clearly observed up to the room temperature in the smaller devices evidenced by the non-monotonical γ vs. T curves. According to Eq. (1) in the main text, γ depends on the cross-sectional of Hall bar S. For convenient comparison, $\gamma' = \gamma S$ is defined with $S = wd$. It is seen that the nonreciprocal coefficient γ increases by ~7 times with the ~10-fold decreasing the cross-sectional area of Hall bar. That is, γ' almost remain unchanged in the Hall bars with different sizes. The experimental results well support the above conclusion.

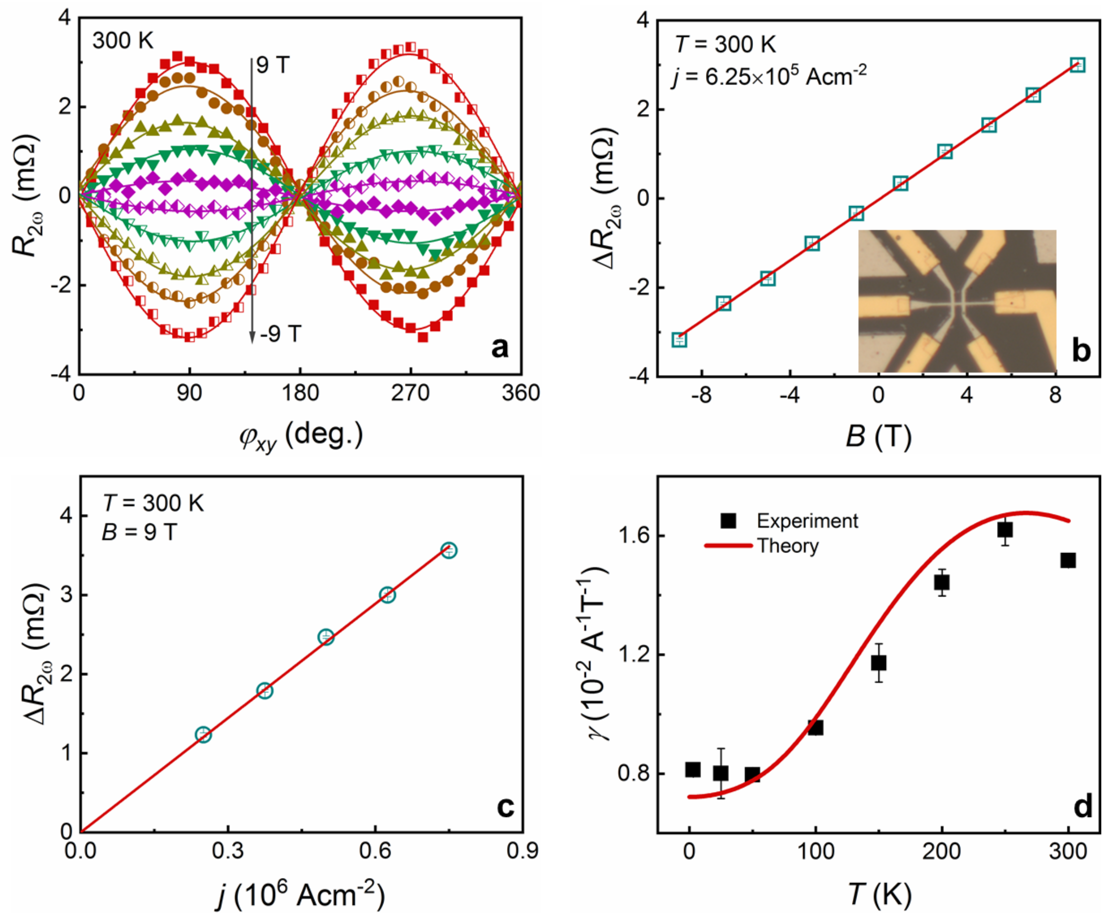

Supplementary Fig. 16 Nonreciprocal charge transport in α-GeTe (sample 5). a In-plane angle-dependent second-order harmonic longitudinal resistance ($R_{2\omega}$) at various magnetic fields measured at 300 K in α-GeTe with a thickness of 64 nm (sample 5). **b** Magnetic field dependent $\Delta R_{2\omega}$ extracted from **a** with $j = 6.25 \times 10^5\ Acm^{-2}$.The inset is the optical image of

the Hall device with dimension of $0.5\,\mu m \times 1.5\,\mu m \times 64\,nm$. **c** $\Delta R_{2\omega}$ as a function of the amplitude of the applied ac current at 300 K and 9 T. **d** Temperature dependent nonreciprocal coefficient γ with the theoretical predication.

Reference

1. He, P. *et al.* Observation of Out-of-Plane Spin Texture in a SrTiO3(111) Two-Dimensional Electron Gas. *Phys. Rev. Lett.* **120**, 266802 (2018).
2. Ideue, T. *et al.* Bulk rectification effect in a polar semiconductor. *Nat. Phys.* **13**, 578 (2017).
3. Dybko, K. *et al.* Experimental evidence for topological surface states wrapping around a bulk SnTe crystal. *Phys. Rev. B* **96**, 205129 (2017).
4. Kremer, G. *et al.* Unveiling the complete dispersion of the giant Rashba split surface states of ferroelectric α−GeTe(111) by alkali doping. *Phys. Rev. Research* **2**, 033115 (2020).
5. Deringer, V. L., Lumeij, M. & Dronskowski, R. Ab Initio Modeling of α-GeTe(111) Surfaces. *J. Phys. Chem. C* **116**, 15801 (2012).
6. Liebmann, M. *et al.* Giant Rashba-Type Spin Splitting in Ferroelectric GeTe(111). *Adv. Mater.* **28**, 560 (2016).
7. Elmers, H. J. *et al.* Spin mapping of surface and bulk Rashba states in ferroelectric α-GeTe(111) films. *Phys. Rev. B* **94**, 201403 (2016).
8. Kriegner, D. *et al.* Ferroelectric Self-Poling in GeTe Films and Crystals. *Crystals* **9**, 335 (2019).
9. Polking, M. J. *et al.* Size-dependent polar ordering in colloidal GeTe nanocrystals. *Nano Lett.* **11**, 1147 (2011).
10. Wang, X. *et al.* Self-screening induced abnormal stability of ferroelectric phase in GeTe ultrathin films. *Appl. Phys. Lett.* **113** (2018).
